# Reflection’s role in learning: increasing engagement and deepening participation

**DOI:** 10.1007/s40037-016-0296-y

**Published:** 2016-09-14

**Authors:** Karen V. Mann

**Affiliations:** Division of Medical Education, Faculty of Medicine, Dalhousie University, Halifax, Nova Scotia Canada

It’s a pleasure to write a commentary that has as its basis a study which examines the impact of reflection on learning. As one considers the evolution of our understanding of reflection and its place in health professions education, several observations emerge. Educators have understood reflection as a key element of learning for decades, dating at least back to John Dewey [1]. However, its place in medical education has emerged more recently. Donald Schön’s [2, 3] description of how learning from experience occurred in practice introduced a new epistemology of learning to medical education.

For some time, attempts at introducing reflection into medical and health professions education curricula ran separately from and parallel to what were regarded as the central curricular elements [4] and challenges existed for their integration [5]. Pedersen [6], in a review of empathy teaching, noted the dichotomy of science and humanities in the world of physicians. He suggests that the effect of learning biomedical skills and knowledge, combined with an unquestioned reliance on ‘objectivity’ of biomedical knowledge, may not allow for or recognize the importance of deriving meaning and interpretation, such as through reflection. Hence it may remain on the periphery.

How our ideas have changed! Reflection is now regarded as an essential capacity fundamental to self-regulation and learning. Accumulating evidence demonstrates many benefits: it is integral to self-assessment, enhances acceptance of feedback, allows for the integration of new and existing knowledge and experience, facilitates reconciliation of cognitive and affective elements of experience, supports the development of professional identity, and enhances diagnostic accuracy [7].

The challenge facing educators now is a different one: How can reflective practice be integrated into the core of medical education? How can its development be effectively scaffolded and structured?

Larsen et al. [8] describe a reflective learning approach in which learners in the clinical setting engaged in daily reflections. Their goal was to understand the impact frequent reflection-on-action might have on priming learners’ reflection-in-action: i. e. awareness of their thoughts and actions in the moment. In the authors’ view this could support the development of a habit of mind of situational awareness and monitoring. They also explored the effect of frequent reflection on the remembering of experience, drawing on principles and evidence from cognitive science about the effect and timing of recall on retention.

The findings of this pilot study both offer interesting insights and raise questions about how reflection may enhance learning. A practical consideration is the feasibility of frequent reflection as an educational activity. As the authors note, and others have shown, feedback on reflection is an essential component of the learning that results. Providing such feedback requires the commitment of faculty teachers; it also requires faculty development and support to enhance the likelihood that feedback will lead to improved ability to reflect, and beyond the current experiences.

The relationship between reflection-on-action and reflection-in-action is also noteworthy. Larsen et al.’s findings support that students may find themselves being more aware in the moment, as a means of preparing for and gathering data for their later reflection-on-action. Whether or how reflection-on-action stimulates reflection-in-action or situational awareness is as yet unclear. It seems that these are two separate types of reflection, and with different purposes. Reflection-in-action suggests that adjustments are made in the moment, whereas reflection-on action may lead to adjustments to future learning and actions. This seems an area for further exploration.

It is also useful to consider the affective component of reflection. While Schön’s [2, 3] model was less explicit about this aspect, other models more clearly elicit the importance of reflecting on both thinking and feeling [4]. Larsen and colleagues [8] have focused on the reflection process rather than the specific content of the reflections; there is no explicit mention of emotions or feelings in the survey questions to which the students responded. If we as educators are silent about emotion could we unintentionally reinforce the idea that Wald [7] describes, of reflection on feelings as an ‘optional extra’? Recognizing how thinking and feeling may interact and affect each other can contribute both to enhanced self-awareness and to the development of professional identity. Learners may need structure and support to reflect in this way.

More broadly, approaches to understanding learning may be helpful in situating the role of reflection. Sfard [9] coined the metaphors of ‘acquisition’ and ‘participation’ to describe learning. The *Acquisition* metaphor describes individual learning and development of knowledge, skills and values that are then applied to and underlie practice. The *Participation* metaphor sees learning as occurring in active participation in the activities of the community one desires to enter, learning about the actual practice of the profession. Participation involves developing an understanding of one’s role in the community, socialization into the profession and development of professional identity as an increasingly responsible and contributing member of the community. Connecting and integrating these experiences and constructing one’s professional identity requires meaning-making, an activity that requires reflection.

Billet’s concept of ‘readiness to learn’ may also be helpful. Billett [10, 11] writes about learning from work, or practice-based learning. He emphasizes the importance of readiness to learn, by which he means learners’ readiness to engage with the experiences they encounter. Developing competence occurs in conceptual, procedural and dispositional areas: that is, learners develop in what they know, can do, and value. Key to learning effectively from work is the learner’s active engagement. Billett positions learners as making meaning and constructing connections among the things they are learning; they do so based on their previous experience. Therefore, two learners may have a similar experience, but they will each learn something different from it. In the context of Billett’s explanation, reflection as described by Larsen et al, [8]. can play an important role in several aspects of learning from practice: it promotes active engagement and making meaning of experience; it can increase intentionality, and it also focuses learners on using their reflections as a means to guide their future learning, and therefore to be more goal-directed.

Larsen and colleagues [8] have provided valuable insights into learners’ experience with daily reflections. Their findings show promising potential effects on learning of frequent reflection on experience. As educators and researchers, how can we build on their findings using the understandings contributed by both Sfard [9] and Billet [10, 11]? Can we provide experiences with which students can engage and in which they can participate? Importantly, in so doing, can we view reflection as a means of engagement which supports learners’ readiness – their development of competence in what they know, can do and value, and as a vehicle for deepening participation?
